# The Hydro-Electric Methods in Medicine

**Published:** 1893-06

**Authors:** 


*The Hydro-Electric Methods in Medicine.* By W. S. HEDLEY, M.D. Pp. x., 156. London: H. K. LEWIS. 1892.

From the middle of the eighteenth century, when the first systematic attempts were made to employ electricity as a therapeutic agent, to the end of the nineteenth, is a considerable period in the history of modern medicine; yet we are only just beginning to see our way through the darkness which envelops the subject of electro-therapeutics.

The belief that nervous impulses were really electrical phenomena not unnaturally led us to hope that electricity, if not actually "life," constituted a very essential element in nervous functions, and originated the earlier expectations of great things, which unfortunately were never realised outside the imaginations of hysterical or deluded victims of quackery.

But electro-therapeutics has been developed on more scientific and successful lines, and a knowledge of its methods is daily becoming more essential to practitioners who wish to be in touch with the latest resources of their art.

The book before us does not pretend to be a treatise, nor is it a handbook, but merely a collection of electro-medical papers, "in groups," the first of which gives it a name. It gives detailed information on the methods of administering the hydro-electric baths, and many useful hints on applied electro-therapeutics, which will be interesting to those who have previously mastered the elements of medical electricity; but for those unversed in electrical terms it is rather technical and disconnected, "its chief aim being to examine certain questions from some special, perhaps rather neglected, standpoint."

